# Marine Sponges in a Snowstorm – Extreme Sensitivity of a Sponge Holobiont to Marine Oil Snow and Chemically Dispersed Oil Pollution

**DOI:** 10.3389/fmicb.2022.909853

**Published:** 2022-07-15

**Authors:** Johanne Vad, Laura Duran Suja, Stephen Summers, Theodore B. Henry, J. Murray Roberts

**Affiliations:** ^1^Changing Oceans Group, School of Geosciences, The University of Edinburgh, Edinburgh, United Kingdom; ^2^Singapore Centre for Environmental Life Sciences Engineering, Nanyang Technological University, Singapore, Singapore; ^3^School of Energy, Geoscience, Infrastructure and Society, Heriot-Watt University, Riccarton, United Kingdom

**Keywords:** holobiont, marine snow, marine oil snow, metatranscriptomics, marine sponges, *Halichondria panicea*

## Abstract

Holobionts formed by a host organism and associated symbionts are key biological units in marine ecosystems where they are responsible for fundamental ecosystem services. Therefore, understanding anthropogenic impacts on holobionts is essential. Sponges (Phylum Porifera) are ideal holobiont models. They host a complex microbial community and provide ecosystem services including nutrient cycling. At bathyal depths, sponges can accumulate forming dense sponge ground habitats supporting biodiverse associated communities. However, the impacts of spilled oil and dispersants on sponge grounds cannot be understood without considering exposures mediated through sponge filtration of marine snow particles. To examine this, we exposed the model sponge *Halichondria panicea* to oil, dispersant and “marine oil snow” contaminated seawater and elucidate the complex molecular response of the holobiont through metatranscriptomics. While the host response included detoxification and immune response pathways, the bacterial symbiotic response differed and was at least partially the result of a change in the host environment rather than a direct response to hydrocarbon exposure. As the sponge host reduced its pumping activity and internal tissue oxygen levels declined, the symbionts changed their metabolism from aerobic to anaerobic pathways possibly via quorum sensing. Furthermore, we found evidence of hydrocarbon degradation by sponge symbionts, but sponge mortality (even when exposed to low concentrations of hydrocarbons) implied this may not provide the holobiont with sufficient resilience against contaminants. Given the continued proposed expansion of hydrocarbon production into deep continental shelf and slope settings where sponge grounds form significant habitats it is important that dispersant use is minimised and that environmental impact assessments carefully consider the vulnerability of sponge holobionts.

## Introduction

Symbiosis as defined by De Bary (1879) refers to close, long-term and stable associations between organisms of different species. In marine ecosystems, symbiosis between microbes and multicellular organisms is ubiquitous and constitutes a key process driving evolution (Björk et al., 2019; Apprill, 2020). From the concept of symbiosis, the term holobiont emerged in the 1990s and is defined as a host and its associated symbionts forming a single biological unit (Dittami et al., 2021). The term holobiont is now widely used in the field of marine ecology (Dittami et al., 2021) and it is understood that holobionts fulfil important ecosystem services in marine environments (Apprill, 2020). Despite their importance, there is minimal understanding of how marine holobionts respond to environmental stress and how stressors affect the ecosystem services they deliver. In tropical corals (the most studied marine holobiont), environmental stress can lead to coral “bleaching,” the dissociation between the coral host and its microalgal symbionts, ultimately degrading tropical coral reef habitats and highlighting the key role of the symbionts in the holobiont health (e.g., Apprill, 2020). Understanding the impact of environmental stress and pollutants on marine holobionts in poorly understood systems is therefore crucial.

Sponges are ideal holobiont models to study the importance of symbiosis in marine organisms (Pita et al., 2016, 2018; Webster and Thomas, 2016). Sponges are key filter-feeding organisms found in many benthic environments (Van Soest et al., 2012) and host complex microbial communities that actively contribute to holobiont metabolism and health (Pita et al., 2018). Sponge associated microbial communities can account for more than 40% of holobiont biomass (Webster and Taylor, 2012) and contribute to core biological functions including carbon and nitrogen cycling (Hoffmann et al., 2009; Maldonado et al., 2012; Li et al., 2014). Mediated through such close metabolic links, sponge holobionts are integral in benthic ecosystem functioning including bentho-pelagic coupling, i.e., the transfer of energy and nutrients from the benthos to the water column (De Goeij et al., 2013). When present at high densities, sponges form important habitats known as sponge grounds. These habitats are found at all depths and regions, including the polar oceans and support high levels of biodiversity, meeting the criteria as both vulnerable marine ecosystems (VMEs) by the UN Food and Agriculture Organisation and as ecologically and biologically significant marine areas (EBSAs) by the Convention on Biological Diversity (Maldonado et al., 2015). But despite their ecological significance and vulnerability, the impacts of pollutants on sponge holobionts remain poorly understood.

Offshore hydrocarbon exploration and production activities are taking place in areas where sponge grounds are present (Williams et al., 2018; Kazanidis et al., 2019; Vad et al., 2019), yet the potential impacts of an oil spill on sponges are not fully understood. During an oil spill, dispersants are often applied to reduce the formation of surface slicks by increasing the dissolution of hydrocarbons into the water column (National Research Council [NRC], 2005). For example, during the Deepwater Horizon oil spill which took place in the Gulf of Mexico in 2010, a record seven million litres of dispersants were applied both at the surface and near the well (Girard and Fisher, 2018). So far, only two studies have investigated the impacts of hydrocarbons and dispersant exposures on adult sponges and sponge larvae (Luter et al., 2019; Vad et al., 2020). Both studies produced water accommodated oil fractions (WAFs – mixtures of oil in seawater) and chemically enhanced WAFs (CEWAFs – mixtures of dispersant and oil in seawater) to expose sponges to hydrocarbons and dispersant. Both studies showed significant changes in sponge gene expression profiles (Luter et al., 2019; Vad et al., 2020). There is also anecdotal evidence that sponges host oil-degrading bacteria (Rubin-Blum et al., 2017). However, no study yet has investigated whether these symbionts provide enhanced resilience to oil contamination. Even more importantly, there are no studies that have examined the effect of oil and dispersant on “marine snow,” a highly significant nutritional source actively filtered and ingested from seawater by sponges.

Marine snow (MS) is composed of organic and inorganic material, as well as prokaryotic and phytoplankton cells (Simon et al., 2002). It has a pivotal role in the transport of organic material from the upper reaches of the water column as it settles to the seafloor (Simon et al., 2002). At the seafloor, marine snow forms a critical food source for many benthic filter and suspension-feeders (Newell et al., 2005). Marine oil snow (MOS), obtained when small oil droplets get enclosed in marine snow aggregates (Duran Suja et al., 2017), was first observed during the Deepwater Horizon oil spill (Passow et al., 2012) during which MOS was detected within 2 weeks of the spill (Fu et al., 2014). While MOS has also been successfully produced in laboratory experiments by mixing crude oil, dispersant and seawater (Fu et al., 2014; Duran Suja et al., 2017), the impact of MOS on benthic organisms is not well characterised. One laboratory study found that MOS significantly reduced the survival of macro-invertebrates through exposure to oil and hypoxic conditions (Van Eenennaam et al., 2018). Cold-water corals were significantly affected by MOS in the field following the Deepwater Horizon oil spill, displaying a strong stress response at molecular level and visual signs of injury with limited recovery even 7 years after the spill (DeLeo et al., 2018; Girard and Fisher, 2018). To date no study has examined the impact of MOS on the sponge holobiont.

We addressed this knowledge gap by conducting an experiment during which the widely distributed intertidal sponge model *Halichondria panicea* (commonly known as the breadcrumb sponge) was exposed to MS, MOS, and CEWAF. Metatranscriptomic sequencing was undertaken after 3 days of exposure to determine the holobiont response to hydrocarbon contamination. *H. panicea* symbiotic community composition is dominated by alphaproteobacterium “*Candidatus Halichondribacter symbioticus”* (Althoff et al., 1998; Wichels et al., 2006; Knobloch et al., 2019), of which the reference genome has been recently investigated (Knobloch et al., 2020). To inform the metatranscriptomic results, treatment properties (pH, dissolved oxygen concentration and hydrocarbon concentrations) were monitored throughout the exposure. In addition, sponge pumping activity was assessed by measuring tissue oxygen concentration depth profiles. We investigated three hypotheses: (1) the sponge holobiont can be exposed to hydrocarbons through consumption of MOS; (2) the response to hydrocarbons is shared between the sponge host and its associated microbial symbionts; and (3) the sponge associated microbial community is capable of hydrocarbon degradation.

## Materials and Methods

### Sampling and Experimental Design

Sponge and seawater samples were collected from the Berwickshire coast of the North Sea as detailed in Supplementary Information (SI). Sponge samples were identified by observation of their spicules under light microscopy and the organisation of their skeleton following nitric acid dissolution of the sponge tissue. A flow-through experimental apparatus was built in which each sponge sample was kept in an individual incubation chamber (as described in Vad et al., 2020 and in the Supplementary Information). Sponges were left in the incubation chambers to acclimatise for 48 h in seawater before the beginning of the experiment. The experimental apparatus was kept in the dark to avoid photo oxidation and in a temperature-controlled room (10°C). At the start of the experiment, header flasks connected to the incubation chambers, were filled with relevant treatment solutions (see next section). The experiment lasted for 7 days, exposure was continuous, and the achieved hydrocarbon concentrations in each treatment were measured on day 1, 3, and 5 of the exposure (see section entitled “Physico-Chemical Characterisation of Treatment Solutions”).

### CEWAF, MS and MOS Preparation

CEWAF solutions were produced following the Chemical Response to Oil Spills: Ecological Research Forum methodology (Singer et al., 2000) with Schiehallion crude oil (BP) and dispersant Slickgone NS (Dasic International). Further details regarding Schiehallion oil and dispersant Slickgone NS are given in the Supplementary Information. In this study, a CEWAF solution (30 L in total) at a nominal oil loading of 1 g of crude oil per L was prepared and Slickgone was applied at a volume ratio of 1:10 as advised by the manufacturers. The mixtures were then mixed with a magnetic stirrer at a speed of 300 rpm (small vortex visible) for 18 h at 10°C in the dark. The mixtures were allowed to stand for 3 h and the aqueous phases (avoiding non-dispersed/solubilised oil or dispersant) were sub-sampled into clean (autoclaved and acid-washed with 5% nitric acid) screw-capped glass bottles with Teflon caps. The CEWAF solutions were stored at 4°C until used in the experiment. MS aggregates were obtained by rotating three bottles of seawater (to allow for enough aggregates to form; as established in preliminary trials) at 10°C throughout the length of the experiment. MOS aggregates were similarly obtained by rotation three bottles of CEWAF solutions at 10°C throughout the duration of the experiment. During preliminary trials, it was established that the MS and MOS aggregates took 2 to 3 days to synthesise, so the preparation of these treatments were started at the same time as the acclimatisation period. Each day during the exposure, three to five MS and MOS aggregates of 2–3 mm across in diameter were gently pipetted out of the bottles and added to the MS and MOS treated incubation chambers.

### Physico-Chemical Characterisation of Treatment Solutions

Seawater pH and dissolved oxygen concentrations were measured in each incubation chamber throughout the experiment. Changes in dissolved oxygen concentrations and pH were detected by performing an ANOVA at each time point after checking for normality and homoscedasticity. Tukey’s all-pair comparisons tests were then used to identify statistical differences (Hothorn et al., 2008; R Core Team, 2021).

Seawater samples (90 mL) were also collected for liquid-liquid hydrocarbon extraction using analytical grade dichloromethane. Following extraction, samples were sent to Terra Tek for speciated USEPA PAHs GC-MS analysis targetting naphthalene, acenaphtylene, acenaphthene, fluorene, phenanthrene, anthracene, fluoranthene, pyrene, benzo[a]anthracene, chrysene, benzo[b]fluoranthene, benzo[k]fluoranthene, benzo[a]pyrene, indeno[1,2,3-cd]pyrene, dibenzo[ah]anthracene and benzo[ghi]perylene. One millilitre of extracts was spiked with internal standard in a 2 mL vial, capped and analysed. The GC-MS analysis was performed on an Agilent 6890 GC with 5973 inert MSD detector in SIM mode, on a 30 m × 0.25 mm ID × 0.25 μm df column with 5 m guard column (Restek p/n 13623-124). PERMANOVA analysis (Oksanen et al., 2020) were applied to test for statistical differences in hydrocarbon concentrations between treatments and time points.

### Sponge Tissue Dissolved Oxygen Concentration Profile

Dissolved oxygen concentration was measured within sponge tissues as a proxy for pumping activity (Hoffmann et al., 2008) using a Presens needle spot mounted on a Presens micromanipulator. Measurements were taken at the sponge’s surface and then every 100 μm within its tissue. Three profiles were measured for each sponge at every time point and each profile location was chosen randomly on each sponge. At every time point, statistical differences in dissolved oxygen concentration through the sponge tissue depth between treatments were determined by constructing repeated-measures linear mixed effect models where oxygen concentration was the response variable and depth and treatment were the explanatory variables (Pinheiro et al., 2021).

### RNA Extraction and Sequencing

Total RNA was extracted from sponge tissue samples collected after 3 days of exposure and preserved in RNA later at −20°C. Extractions were performed using Qiagen Total RNA Blood and Tissue extraction kits following manufacturer’s instructions. DNA was removed from the extractions using Qiagen DNase kits and RNA extracts were eluded into 30 μL of DNA/RNA free sterile water. RNA quality and quantity were then, respectively, assessed by spectrophotometer using a NanoDrop and by fluorometer using a Qiagen Qubit kit. Only RNA samples with 260/230 and 260/280 ratios between 1.8 and 2.2 were submitted for sequencing. One sample (MS3) failed this quality check and was therefore not sent to sequencing. RNA extractions were sent to Edinburgh Genomics for library preparation and NovaSeq 100PE sequencing. Library preparation was performed by completing a RiboZero treatment with prokaryotic and eukaryotic rRNA depletions. Paired-end reads were run to 100 bp to yield at least 750 M + 750 M reads.

### Metatranscriptomic Analysis

When sequencing data were received, read quality was assessed using FastQC (v0.11.9; Andrews, 2015). Short (<75 bp) and low-quality sequences (Phred score < 30) were removed using Cutadapt (v3.1; Martin, 2011). Reads from all samples were then assembled *de novo* with Megahit (v1.2.9 with default settings; Li et al., 2015) on the University of Edinburgh Linux Compute Cluster Eddie. Short contigs (<300 bp) were removed from the meta-assembly and the meta-assembly was then annotated with Diamond Blast (v2.0.9 with default settings; Buchfink et al., 2021) against the nr database (accessed in November 2020). An *E* value cut-off of 1e-5 was used to select the best blast hits. Bowtie2 (v2.4.2) was used to align the reads back to the meta-assembly with setting “sensitive local” (Langmead and Salzberg, 2012). Raw and normalised transcript abundance was calculated using RSEM with default settings (v1.3.2; Li and Dewey, 2011). Taxonomic identification of the meta-assembly contigs was assessed using MEGAN (v6.21.2; Huson et al., 2016) to separate sponge (host) and bacterial (symbionts) assemblies. In addition, presence of key alphaproteobacterium symbiont “*Candidatus Halichondribacter symbioticus”* was assessed by mapping all reads to the bacterial reference genome (Knobloch et al., 2019) with Bowtie2 with setting “sensitive local” (v2.4.2). Differential expression analysis was then performed on the sponge and symbiotic dataset separately in R using DESeq2 (v3.13; Love et al., 2014). Differentially expressed genes compared to the control group were identified at a FDR adjusted value of 0.01. KEGG annotations were obtained with GhostKoala (Kanehisa et al., 2016; accessed in January 2021). To support the differential expression analysis, bacterial metagenomes were compared between all treatments. Prokka (v1.14.5 with default metagenome settings) was first used to convert bacterial transcripts obtained in each treatment to protein sequences (Seemann, 2014). MicrobeAnnotator (v2.0.5 with diamond method; Ruiz-Perez et al., 2021) was then used to annotate the proteins through searches in the KoFamscan, UniProt Swissprot, RefSeq and Trembl databases (last accessed in May 2022). This enhanced annotation enables an in-depth comparisons of metagenome completeness. Further details on how figures were generated for this manuscript is provided in the Supplementary Information.

## Results

### Gross Observations

**FIGURE 1:**
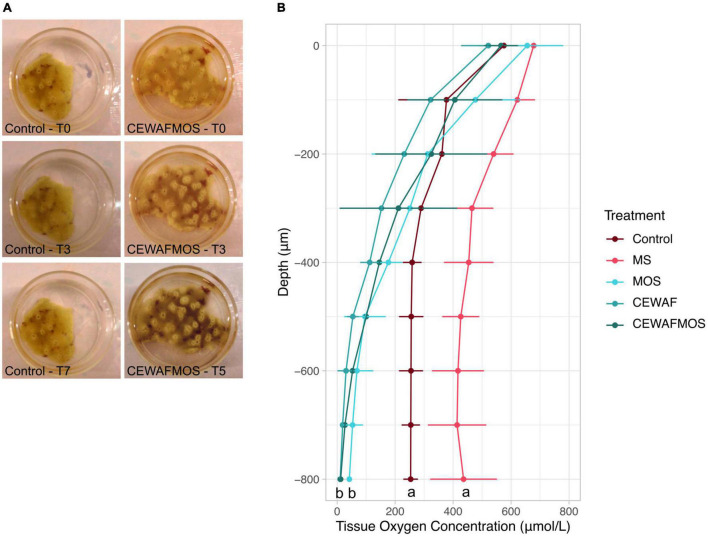
**(A)** Change in tissue colouration in the CEWAFMOS treatment compared to control over time. **(B)** Tissue oxygen concentration (μmol/L) across sponge tissue depth (μm) in each treatment conditions after 3 days of exposure. Note that similar profile was obtained with measurements taken after 5 days of exposure (no statistically significant differences were found between time points within each treatment). Different lowercase letters indicate a significant difference between treatments.

Sponges in the control and MS treatment retained their bright yellow colour and appeared healthy throughout the experiment (7-day exposure). In the MOS, CEWAF and CEWAF + MOS treatments, sponge samples slowly turned grey and dark and eventually died (Figure 1A). Samples in the CEWAF + MOS and CEWAF treatment all died after 5 days of exposure while samples exposed to MOS alone died after 7 days.

### Physicochemistry of Treatments

Overall, seawater dissolved oxygen concentration and pH remained constant in the control, MS and MOS treatment throughout the experiment (Supplementary Figure 1). Dissolved oxygen concentration remained above 320 μmol/L and pH varied between 7.7 and 8.1 in the control, MS and MOS treatments. In contrast, dissolved oxygen concentration and pH were significantly lower in the CEWAF and CEWAF + MOS at day 1, 3, and 6 of exposure (Supplementary Figure 1). Dissolved oxygen reached a minimum of 256.2 ± 3.8 μmol/L (final day of exposure) and 259.4 ± 14.9 μmol/L (first day of exposure) in the CEWAF and CEWAF + MOS treatment, respectively, while pH decreased to 7.3 ± 0.1 on the final day of the exposure in both treatments.

None of the 16 polycyclic aromatic hydrocarbons (PAHs) measured in this study were detected in the control and MS treatments throughout the experiment. In the MOS, CEWAF, and CEWAF + MOS treatments, only naphthalene, fluorene, and phenanthrene were measured above limits of detection (Supplementary Figure 2). Concentrations of all three hydrocarbons were highest in the CEWAF + MOS treatment where naphthalene concentrations rose to 8.8 ± 5.3 μg/L after 1 day of exposure while fluorene and phenanthrene concentrations, respectively, reached 0.6 ± 0.3 μg/L and 3.8 ± 0.8 μg/L after 1 day of exposure. As in the CEWAF + MOS treatment, naphthalene was the most common hydrocarbon detected in the MOS and CEWAF treatments where the concentration reached a maximum of 0.6 ± 0.1 μg/L and 7.0 ± 2.4 μg/L after 3 days of exposure, respectively, (Supplementary Figure 2). Accordingly, Σ_16_PAHs were highest in the CEWAF + MOS treatment where it rose to 13.3 ± 5.5 μg/L after 1 day of exposure while Σ_16_PAHs peaked at 11.5 ± 2.9 μg/L and 0.7 ± 0.0 μg/L in the CEWAF and MOS treatments after 3 days of exposure, respectively. The PERMANOVA analysis revealed that hydrocarbon concentrations varied significantly between treatment and time points (Supplementary Table 1).

### Sponge Tissue Oxygen Concentration Profiles

Oxygen concentration within the sponge tissue dropped in all treatments with tissue depth and were consistent across all time points considered (Figure 1B). However, significant differences were identified in the sponge oxygen depth profile across treatments. In control conditions, the dissolved oxygen concentration in the sponges stabilised at ∼250 μmol/L at ∼500 μm tissue depth. In the presence of MS, the sponge oxygen depth profile reached a plateau at ∼400 μmol/L at ∼500 μm depth. When exposed to hydrocarbons (MOS, CEWAF or CEWAF + MOS treatments), the sponge oxygen concentrations decreased rapidly with tissue depth and reached 0 from ∼600 μm tissue depth (Figure 1B). Statistically significant differences between oxygen depth profiles were found between the control-MS conditions and the MOS-CEWAF-CEWAF + MOS treatments after both 3 and 5 days of exposure (Figure 1B).

### Metatranscriptomic Analysis

The *de novo* metatranscriptomic assembly was composed of 557,483 contigs, built from a total of 789,274,613 paired sequence reads. The assembly GC content was 40.42% and the assembly N50 reached 1,615 base pairs. Overall, 68.9% of the contigs (383,995 contigs) were identified against the nr database. The annotated sequences included 48,221 eukaryotic (12.6%), 325,636 bacterial (84.8%), 1,331 archaeal (0.34%) and 1,209 viral (0.31%) sequences. In total, 14,791 eukaryotic sequences were identified from poriferan sequences (30.7% of eukaryotic sequences) while 10,747 were derived from cnidarian sequences (22.3% of eukaryotic sequences). Bacterial sequences were dominated by sequences identified in Proteobacteria (175,877 sequences equivalent to 54.0% of bacterial sequences) and in the Fibrobacteres-Chlorobi-Bacteroidetes group (57,251 sequences equivalent to 17.6% of bacterial sequences). Presence of key symbiont “*Ca. H. symbioticus*” was assessed in all samples and overall read alignment scores with the reference genome were consistent across treatments varying between 16.4% ± 0.8 and 20.4% ± 3.6 of all reads (sponge and bacterial; Table 1).

**TABLE 1:** Overall alignment scores of all reads against “*Ca. H. symbioticus*” reference genome in each sample.

Sample	Overall alignment (in%)	Treatment average (in% ± SD)
Co1	18.54	17.4 ± 1.4
Co2	17.86	
Co3	15.92	
MS2	19	17.6 ± 2.0
MS3	16.14	
MOS1	24.29	20.4 ± 3.6
MOS2	17.3	
MOS3	19.58	
CEWAF1	17.33	16.4 ± 0.8
CEWAF2	15.7	
CEWAF3	16.19	
CEWAFMOS1	16.06	16.6 ± 2.7
CEWAFMOS2	19.49	
CEWAFMOS3	14.27	

**FIGURE 2:**
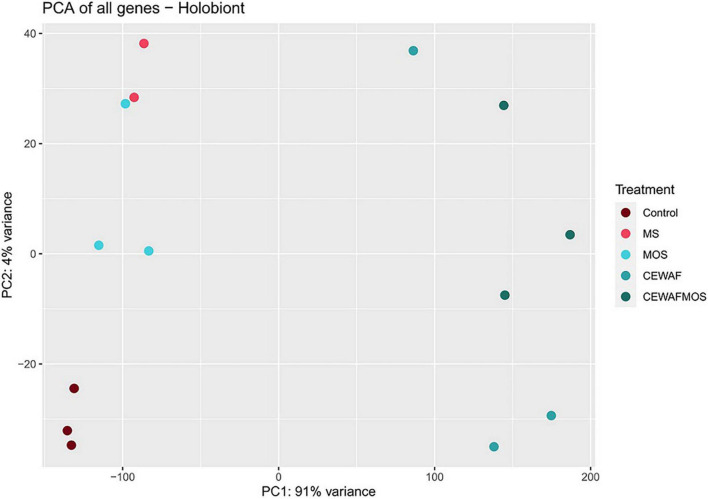
Principal component plot of metatranscriptome-wide expression profiles in *H. panicea* samples exposed to control, MS, MOS, CEWAF, and CEWAF + MOS conditions.

**FIGURE 3:**
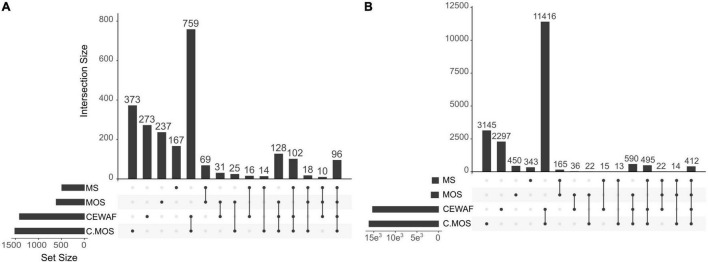
Upset plots showing the number of differentially expressed genes in **(A)** the sponge host transcriptome and **(B)** the symbiotic bacterial metatranscriptome across treatments relative to control conditions. The abbreviation C.MOS stands for CEWAF + MOS. The nodes below each bar plot illustrates which treatments are compared. The bar plot therefore show the number of differentially expressed genes relative to control conditions common to the treatments being compared.

In this study, the holobiont expression profiles changed significantly depending on treatment condition. The first component of the PCA analysis, which explained 91% of the variation in expression profiles, separated the CEWAF and CEWAF + MOS exposed sponges from samples exposed to other conditions (Figure 2). The second component of the PCA analysis explained 4% of the variation in expression and separated control samples from sponges exposed to MS and MOS (Figure 2). The largest number of differentially expressed genes (DEGs) were identified in the CEWAF + MOS treatment in the sponge host and bacterial associated community (Figure 3). Overall, the majority of DEGs were found across the CEWAF and CEWAF + MOS treatments.

**FIGURE 4:**
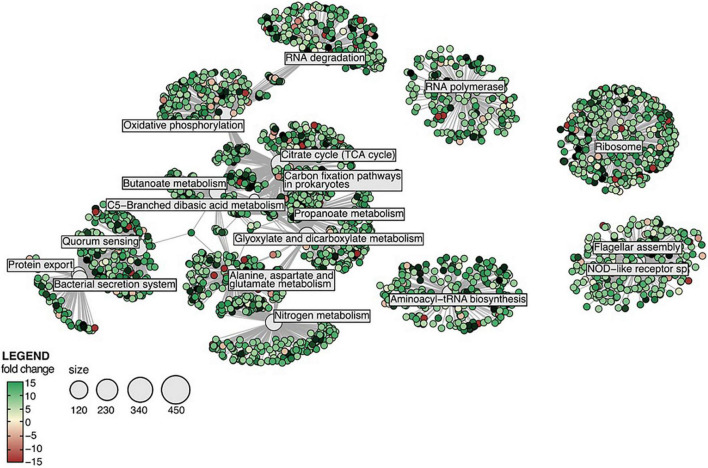
Networks of significantly enriched KEGG pathways amongst the differentially expressed genes in the bacterial symbionts exposed to CEWAF + MOS. Note that KEGG enrichment results for the CEWAF treatment were similar.

Several Kyoto Encyclopaedia of Genes and Genomes (KEGG) pathways were significantly enriched amongst sponge and bacterial DEGs. In the sponge host, several core signalling pathways (NF-kappa B, IL-17, NOD-like receptor, TNF and RIG-I-like receptor) were found significantly enriched in samples exposed to all treatments tested in this study (Supplementary Figure 3). Furthermore, the KEGG pathways “fatty acid biosynthesis” and “biotin metabolism” were significantly enriched in sponges exposed to MS and MOS (Supplementary Figure 3). In the CEWAF and CEWAF + MOS treatment, KEGG pathways involved in the MAPK signalling pathway, apoptosis, and tissue organisation (cell adhesion molecules and Hedgehog signalling pathways) as well as immune system (antigen processing and presentation) were found significantly enriched (Supplementary Figure 3). Amongst the bacterial DEGs, KEGG pathways involved in flagellar assembly and NOD-like receptor signalling pathways were found significantly enriched in all four treatments (Supplementary Figures 4, 5). In the MS treatment, many DEGs were involved in nitrogen metabolism while in the MOS treatment, DEGs contributed to the bacterial secretion system (Supplementary Figure 4). The KEGG pathways enrichment networks produced from the bacterial genes in the CEWAF and CEWAF + MOS treatment were almost identical and bacterial DEGs found in these two treatments contributed predominantly to amino acid synthesis, the carbon cycle and quorum sensing (Figure 4 and Supplementary Figure 5). Although KEGG functions associated with hydrocarbon degradation were not significantly enriched, evidence for hydrocarbon degradation was nevertheless identified amongst the bacterial DEGs pool. In total, 134 DEGs linked to hydrocarbon degradation functions were detected and these genes were significantly up-regulated in samples exposed to the CEWAF and CEWAF + MOS treatments (Supplementary Figure 6).

To further explore the changes in metabolic activities found through differential expression analysis, metagenome annotation and comparisons were performed through Prokka and MicrobeAnnotator (Supplementary Figure 7). Overall, module completeness significantly increased in all treatments compared to controls, including for core metabolic activities such as amino acid synthesis, aromatics degradation, carbon fixation and nitrogen metabolism amongst others (Supplementary Figure 7).

## Discussion

### Scale of Holobiont Response Depends on Hydrocarbon Concentrations

Exposure to MOS alone led to a muted but detectable response in the sponge holobiont compared to the significant changes found in gene expression profiles of sponges in the CEWAF and CEWAF + MOS treatments. This is likely due to hydrocarbon concentrations in the MOS treatment being low throughout the exposure, as only three to five marine oil snow aggregates of 2–3 mm diameter were added to the incubation chambers during each day of the exposure. In fact, Σ_16_PAHs concentrations measured in the MOS treatment (ΣPAH_16_ 0.131 to 0.712 μg/L) were below the range observed (ΣPAH_16_ 0.88 to 6.28 μg/L) in the field during the Deepwater Horizon oil spill (Diercks et al., 2010). Furthermore, the metatranscriptomic profiles of the sponge holobionts exposed to MOS alone were similar to the profiles of sponges exposed to MS. Evidence of sponge feeding was seen in both treatments as KEGG functions such as fatty acid biosynthesis were found significantly enriched amongst the host DEGs pool. Sponge feeding in the MOS and MS treatment is also indicated by the bacterial metatranscriptomic profile. Indeed, the KEGG function flagellar assembly identified as significantly enriched in the bacterial DEGs pool, could, in fact, be indicative of the regeneration of pumping cells (choanocytes) in the sponge tissue. Choanocytes, which are equipped with flagella and constitute the pumping engines of the sponge, are organised in an epithelium known as the choanoderm along a sponge’s internal pumping chambers (De Goeij et al., 2009). These choanocytes are replaced approximately every 6 h to support pumping activities – a rapid cell cycle that matches rates seen in unicellular rather than multicellular organisms (De Goeij et al., 2009). Evidence of feeding in our experiment means that intracellular exposure to hydrocarbons through MOS is therefore highly likely. While only small quantities of MOS aggregates were added to the MOS and CEWAF + MOS treatments, large quantities of MOS were observed *in situ* following the Deepwater Horizon oil spill (Passow et al., 2012; Halanych et al., 2021). Following the well blowout, benthic species including cold-water coral colonies were observed completely covered in MOS aggregates (DeLeo et al., 2018). Despite the MOS treatment in this study prompting only a relatively small metatranscriptomic response in the sponge holobiont, exposures to higher quantities of MOS (and so higher hydrocarbon concentrations) could lead to stronger changes in gene expression profiles, akin to those seen here in the CEWAF and CEWAF + MOS treatments.

### Host VS Symbiont Responses to Hydrocarbon Exposure

This study is the first to apply metatranscriptomics to reveal how sponge symbionts alter their metabolic activities in response to changes in the host tissue as an indirect consequence of environmental pollution. In the CEWAF and CEWAF + MOS treatments, the response to the hydrocarbon exposure between the sponge host and its associated microbial community differed. The KEGG functions found enriched in the host DEG pool highlighted the reliance of the sponge on detoxification, oxidative stress response and immune response pathways. Specifically, the MAPK signalling pathway was found to be a core component of the sponge molecular response to hydrocarbon contamination. This is in line with what has already been described in other marine invertebrates (e.g., Jenny et al., 2016; Park et al., 2020), other sponge species (Châtel et al., 2011) and in *H. panicea* (Vad et al., 2020). In addition, we detected evidence of sponge host tissue degradation as cell adhesion function and the hedgehog signalling pathway were both significantly increased in the CEWAF and CEWAF + MOS treatments. Indeed, the hedgehog signalling pathway has been identified in the genome of the tropical sponge *Amphimedon queenslandica* and is known to be involved in tissue development (Adamska et al., 2007). However, in our study, we also found that the bacterial response outweighed the host response by a factor of 8 to 10 in terms of number of DEGs detected. As part of the bacterial response, the DEGs identified were involved in key metabolic activities such as nitrogen and carbon fixation as illustrated by the relatively small number of significantly enriched KEGG functions detected. Furthermore, the list of enriched KEGG functions identified revealed a change from aerobic to anaerobic activities. Metagenome comparisons performed through MicrobeAnnotator confirmed the changes in metabolic activities identified by differential gene expression analysis. It is therefore possible that the bacterial community responded strongly to the severe drop in dissolved oxygen concentration inside sponge tissues, a consequence of the host halting its pumping activity when exposed to hydrocarbons. A change in bacterial community composition is likely to have contributed to the shifts in metabolic activities found in our study; however, stable alignment scores against “*Ca. H. symbioticus*” reference genome does not indicate a severe shift in microbial community composition. Additional metagenomics analysis would be required to fully address shifts in microbial community composition.

### The Role of Quorum Sensing in the Sponge Holobiont

In the CEWAF and CEWAF + MOS treatments, quorum sensing played a key role in symbionts’ response. Quorum sensing is a form of cell-cell communication defined as the regulation of gene expression in response to the release of chemical signal molecules (autoinducers) beyond a minimal stimulatory threshold concentration (e.g., Miller and Bassler, 2001). In this study, it is possible that quorum sensing enabled the symbiotic community response leading to dramatic changes in metabolic processes and a rapid switch from aerobic to anaerobic metabolism. Although quorum sensing functions were significantly enriched within the bacterial DEG pool, quorum sensing could facilitate symbiont-symbiont as well as host-symbiont interactions (Shiner et al., 2005). Quorum sensing compounds have already been isolated from sponge associated bacteria (Zan et al., 2012) or from sponge extracts (Batista et al., 2018) and identified in sponge metabolic profiles (Papale et al., 2020). However, to the best of our knowledge, this is the first study to identify the specific role of quorum sensing in the response of a marine holobiont to environmental stress.

### Hydrocarbon Degradation Does Not Lead to Hydrocarbon Resilience in Sponges

Hydrocarbon degradation was identified in this study in sponges exposed to higher concentrations of hydrocarbons (CEWAF and CEWAF + MOS treatment). Oil degrading bacteria are present at low concentration in the marine environment, both in seawater and in sediments (Joye et al., 2016). Hydrocarbon degrading symbionts have also been found in sponges collected from deep-sea hydrocarbon seeps (Rubin-Blum et al., 2017). However, we present the first evidence of hydrocarbon degradation in the intertidal sponge *H. panicea*. Despite this, the sponges in the hydrocarbon treatments (CEWAF, CEWAF + MOS and MOS) all died within 7 days of exposure. This demonstrates that the degradation of hydrocarbon by symbionts may not provide the holobiont with any significant increase in resilience to hydrocarbons pollution. This confirms observations made during the Deepwater Horizon oil spill, reporting high mortality in sponge assemblages following the well blowout (Beyer et al., 2016; McClain et al., 2019).

## Conclusion

Sponge grounds cover up to hundreds of km^2^ and support high level of biodiversity by increasing the three-dimensionality of the local benthos, altering local hydrodynamics and contributing to nutrient cycling (Maldonado et al., 2015). Nevertheless, hydrocarbon production activities are already taking place, or are being proposed, within sponge grounds and remediation strategies in place at those fields include the use of dispersants (BP, 2014; BP and Genesis Oil and Gas Consultants Ltd, 2018). For example, oil production activities in the NE Atlantic Faroe-Shetland Channel are being carried within the Sponge Belt Nature Conservation Marine Protect Area (MPA) designated to protect sponge grounds (BP, 2014; BP and Genesis Oil and Gas Consultants Ltd, 2018; Kazanidis et al., 2019; Vad et al., 2019). Further developments such as the CAMBO oil field, located close to the MPA, are currently being considered by the United Kingdom government and dispersants are still listed in the development environmental impact assessment (Siccar Point Energy E&P Ltd, 2020). In addition, sponge grounds form a crucial habitat in benthic Artic environments (Maldonado et al., 2015), which also hold significant untapped oil and gas reservoirs (USGS, 2008) and new major shipping routes due to the furthering losses of Arctic sea ice (Bambulyak and Ehlers, 2020). Sampling at sponge grounds is difficult as they are often found in the deep-sea and require the use of remotely operated vehicles. In this experimental study, we used intertidal sponge model *H. panicea* as a proxy for deeper species. As the molecular pathways involved in the response of *H. panicea* rely on core molecular function and are well preserved across metazoans (Böhm et al., 2001), we can hypothesise that results on deeper species found at sponge grounds such as *Geodia spp* would be similar. Here, sponges exposed to very low concentrations of hydrocarbons (MOS only treatment) died after 7 days of exposure. Our study therefore demonstrates that by contributing to the production of MOS and increasing the concentrations of hydrocarbons in seawater, the use of dispersants increased the risk posed by hydrocarbon contamination to sponges and should therefore be limited within areas that contain sponge grounds. Furthermore, our study also highlights the importance of considering holobiont in ecotoxicology investigations. Future experiments should therefore aim at understanding how changes in the functioning of key holobionts can lead to shifts in the vulnerable habitats they provide, such as sponge grounds.

## Data Availability Statement

The datasets presented in this study can be found in online repositories. The names of the repository/repositories and accession number(s) can be found below: https://www.ebi.ac.uk/ena, ERP134402.

## Author Contributions

JV and LD ran the experiment, analysed the data, and wrote the first manuscript draft. SS contributed to the metatranscriptomics analysis. TH and JR secured funding for the study. All authors contributed to the conceptualisation of the study and the methodology, reviewed, and edited the manuscript.

## Conflict of Interest

The authors declare that the research was conducted in the absence of any commercial or financial relationships that could be construed as a potential conflict of interest.

## Publisher’s Note

All claims expressed in this article are solely those of the authors and do not necessarily represent those of their affiliated organizations, or those of the publisher, the editors and the reviewers. Any product that may be evaluated in this article, or claim that may be made by its manufacturer, is not guaranteed or endorsed by the publisher.
