# Supplementary material for: Marine Sponges in a Snowstorm – Extreme Sensitivity of a Sponge Holobiont to Marine Oil Snow and Chemically Dispersed Oil Pollution
Source: Front Microbiol. 2022 Jul 15;13:909853. doi: 10.3389/fmicb.2022.909853 (PMC9335075; doi:10.3389/fmicb.2022.909853)
Supplement: Supplementary file 1 [file Data_Sheet_1.docx]

**Supplementary Information – Figures and Tables**

Supplementary Figure S1: pH and seawater dissolved oxygen concentration after one, three and five days of exposure across treatment conditions. Different lowercase letters indicate a significant difference between treatment for each parameter respectively.

Supplementary Figure S2: Concentrations of naphthalene, acenaphtylene, acenaphthene, fluorene, phenanthrene, anthracene, fluoranthene, pyrene, benzo[a]anthracene, chrysene, benzo[b]fluoranthene, benzo[k]fluoranthene, benzo[a]pyrene, indeno[1,2,3-cd]pyrene, dibenzo[ah]anthracene, benzo[ghi]perylene and Σ_16_PAHs after one, three and five days of exposure across treatments.

Supplementary Figure S3: Networks of significantly enriched KEGG pathways amongst the differentially expressed genes in the sponge exposed to (A) MS, (B) MOS, (C) CEWAF and (D) CEWAFMOS.

Supplementary Figure S4: Networks of significantly enriched KEGG pathways amongst the differentially expressed genes in the bacterial symbionts exposed to (A) MS and (B) MOS.

Supplementary Figure S5: Networks of significantly enriched KEGG pathways amongst the differentially expressed genes in the bacterial symbionts exposed to CEWAF.

Supplementary Figure S6: Heatmap of all bacterial DEGs involved in KEGG hydrocarbon degradation pathways. The red-blue color bar shows log fold change of DEGs.


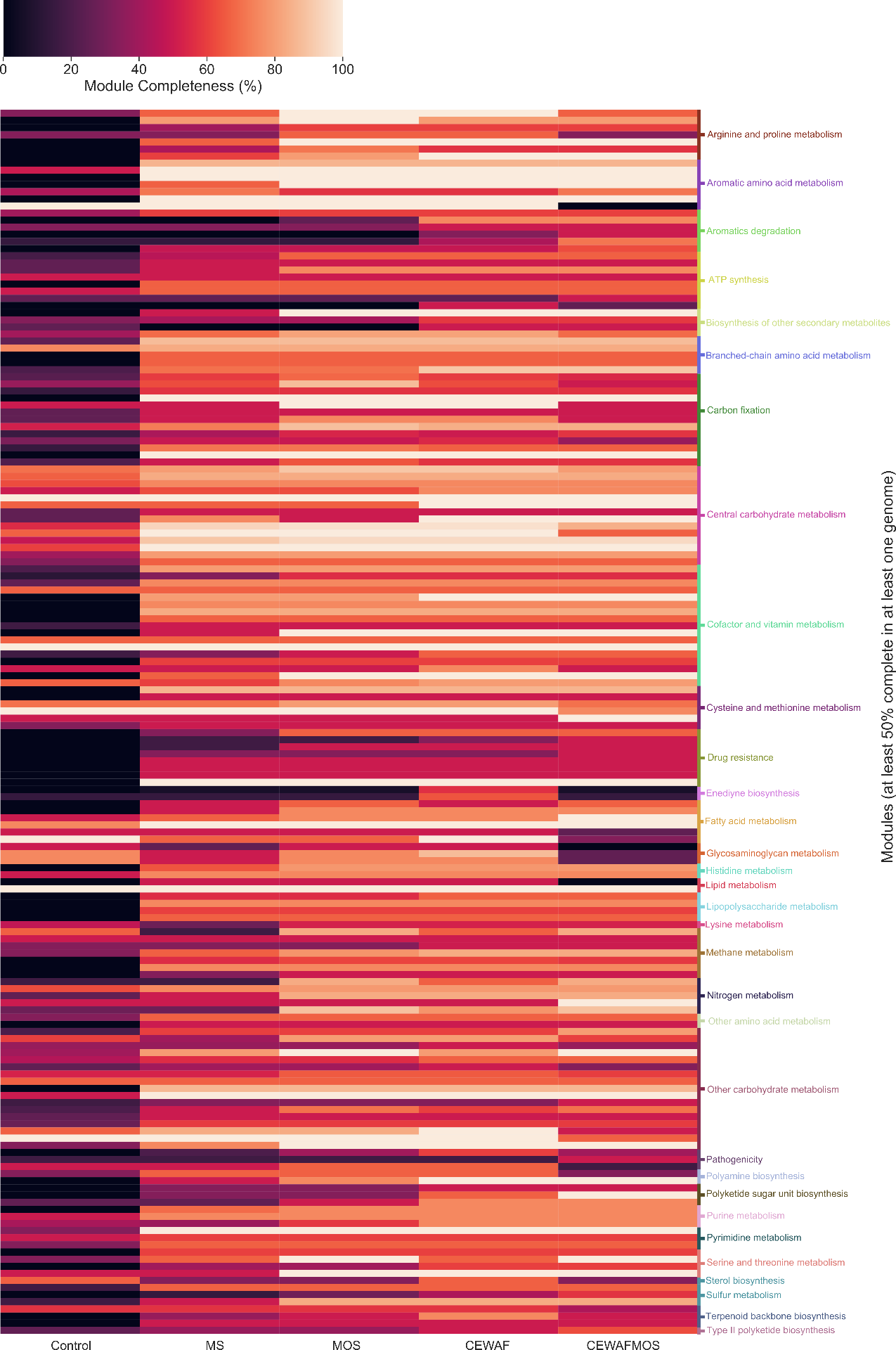


Supplementary Figure S7: Heatmap of bacterial metagenome metabolic module completeness across treatments.

Supplementary Figure S8: Experimental apparatus used in this study with (1) glass header flasks, (2) peristaltic pump, (3) incubation chambers on holding plate and (4) collection reservoir. Figure adapted from Vad et al., (2020).

Supplementary Table 1: Results of PERMANOVA analysis on hydrocarbon concentrations through time and across treatments. Statistically significant results are highlighted in bold.

|  | Degrees of Freedom | Sum of Squares | R^2^ | F statistic | p-value |
| --- | --- | --- | --- | --- | --- |
| Treatment | 2 | 1.661E+08 | 0.423 | 4.823 | **0.005 **** |
| Time | 2 | 2.280E+07 | 0.058 | 0.662 | **0.040*** |
| Treatment*Time Interaction | 4 | 4.854E+07 | 0.124 | 0.705 | 0.710 |
| Residual | 9 | 1.549E+08 | 0.395 |  |  |
| Total | 17 | 3.923E+08 | 1.000 |  |  |

**Supplementary Information – Material and Methods**

*Sampling and experimental design*

In May 2019, sponge and seawater samples were collected at Coldingham bay, located 80 kilometres to the south of Edinburgh (55.89°N, 2.13°W). *H. panicea* sponges can easily be found in the bay at low tide and grows in a fine (1-1.5 mm thick) encrusting yellow to greenish morphotype. Around 20 individuals of *H. panicea* were carefully removed from the rocks with a scalpel and placed into sampling bags filled with freshly collected seawater. Around 50L of surface seawater samples were collected in carboys with the returning tide in clean plastic carboys. All samples were stored in insulated containers and quickly returned to the University of Edinburgh. Upon arrival, sponges were transferred to recirculating seawater tanks in a temperature-controlled room at 10° C (temperature of the seawater in the field). Seawater samples were also stored in the temperature-controlled room.

A flow-through experimental apparatus was used in this study. The experimental apparatus was constituted of 15 individual glass incubation chambers of 750 mL of volume, each equipped with a magnetic stirrer at the top of the chambers. An inflow and outflow in the lid allowed seawater to flow through the chamber at a rate of 750 mL/day (fig. S8). Multi-channel peristaltic pumps were used to control the flow rate through each chamber independently. Each chamber was connected to individual 6 L glass header flasks. PTFE tubing connected each chamber to the peristaltic pumps to limit to a maximum the use of plastic (which interact with hydrocarbons). Gentle air mixing was provided to the water in the header flasks during the whole length of the experiment.

*CEWAF, MS and MOS preparation – oil and dispersant characteristics*

Schiehallion crude oil is characterised by an American Petroleum gravity index of 25.2, a sulphur content of 0.46 % and a viscosity of 67 centistokes (cST) at 20°C (1). Slickgone NS is one of the dispersants approved for use by the United-Kingdom Marine Management Organisation and is listed for potential use in the Faroe-Shetland channel in the case of a spill. Slickgone NS is recommended to be used in a 1:10 dilution dispersant:oil (2, 3).

*Metatranscriptomics analysis – figures*

PCA plots of the expression profiles of all genes within the holobiont were constructed using the R package GGplot2 (4) and RColorBrewer (5). Upset plots of the number of differentially expressed genes identified amongst host and symbionts were drawn using the R package UpSetR (6). ClusterProfiler (7) was used to identified significantly enriched KEGG pathways for each treatment and taxonomic compartment (sponge VS bacterial genes) and pheatmap (8) was used to draw heatmaps of hydrocarbon degradation related genes.

*References*

1. BP Oil International Ltd (2017) *Schiehallion Crude oil from the UK’s Quad 204 Development Transportation and Trade*.
2. BP Oil International Ltd (2014) *Schiehallion Offshore Oil Pollution Emergency Plan and Justification Document*.
3. Marine Management Organisation (2018) *Approved oil spill treatment products*.
4. Wickham, H. (2016) ‘ggplot2: Elegant Graphics for Data Analysis’, Springer-Verlag, New York.
5. Neuwirth, E. (2014) ‘RColorBrewer: ColorBrewer Pallets. R package version 1.1-2. https://CRAN.R-project.org/package=RColorBrewer.
6. Conway, J. R., Lex, A. and Gehlenbord, N. (2017) ‘UpSetR: an R package for the visualization of intersecting sets and their properties’, Bioinformatics, 33(15), pp. 2938-2940.
7. Yu, G., Wang, L., Han, Y. and He, Q. (2012) ‘clusterProfiler: an R package for comparing biological themes among gene clusters’, OMICS: A Journal of Integrative Biology, 16(5), pp. 284-287.
8. Kolde, R. (2018) ‘pheatmap: Pretty Heatmaps’, R package version 1.0.12.
